# Supplementary material for: Comparison of Functional Outcomes between Supervised Rehabilitation and Telerehabilitation in Female Patients with Patellofemoral Pain Syndrome during the COVID-19 Pandemic
Source: Int J Environ Res Public Health. 2023 Jan 26;20(3):2233. doi: 10.3390/ijerph20032233 (PMC9915527; doi:10.3390/ijerph20032233)
Supplement: Supplementary file 1 [file ijerph-20-02233-s001.zip › Stretcing & Exercise program_supplementary file.pdf]

Supplementary Materials File S1: Exercise program

1. Stretching program

**Exercise**	**Implementation**	**Duration**
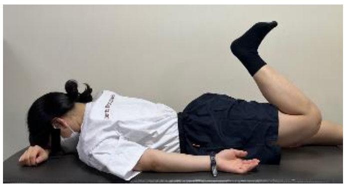<br>- Dynamic stretching of the quadriceps	-In the prone position, hip extension with maximally flexed knee joint.<br>(15 repetitions, 3 sets)	
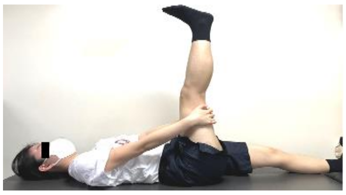<br>-Dynamic stretching of the hamstring	-Repeatedly performing the movement of 90˚ hip joint flexion in the supine position and 90˚ knee joint flexion position, extending the knee to the end of the joint range of motion and holding it for 1 second<br>(15 repetitions, 3 sets)	15 minutes
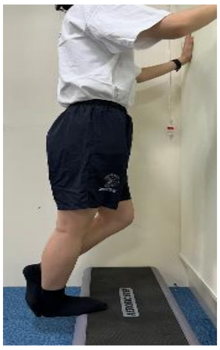<br>-Dynamic stretching of the gastrocnemius	-In a standing position, the involved leg is straightened, and then dropping the heel down toward the floor.<br>(15 repetitions, 3 sets)	

2. Open and closed kinetic chain exercises

Exercise	Progress	Duration
**Open kinetic chain exercises**		
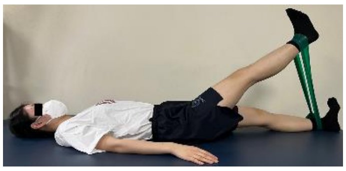<br>- Straight leg raising	- Weeks 1-2: 10 reps, 2 sets, hold for 5 seconds<br>- Weeks 3-4: 12 reps, 3 sets, hold for 10 seconds (resistance: green)	
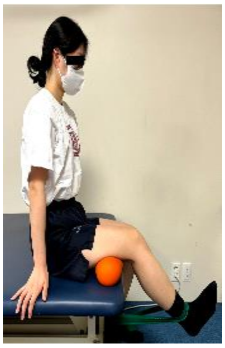<br>- Knee extension	- Weeks 1-2: 10 reps, 2 sets, hold for 5 seconds<br>- Weeks 3-4: 12 reps, 3 sets, hold for 10 seconds<br>- Weeks 5-6: additional resistance band applied to the weeks 3-4 (resistance: green)	
**Closed kinetic chain exercises**		
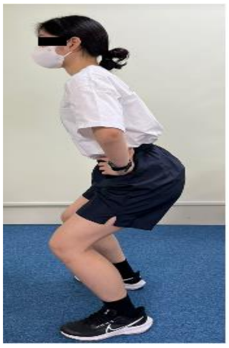<br>- Double leg squat	- Weeks 1-2: 10 reps, 2 sets, hold for 5 seconds<br>- Weeks 3-4: 12 reps, 3 sets, hold for 10 seconds<br>- Weeks 5-6: additional resistance band applied to the weeks 3-4 (resistance: green)	35 minutes

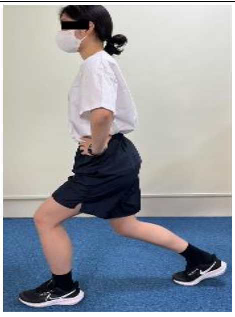

- Forward lunge

- Weeks 1-2: 12 reps, 3 sets
- Weeks 3-4: 12 reps, 3 sets
- Weeks 5-6: additional resistance band applied to the weeks 3-4 (resistance: green)

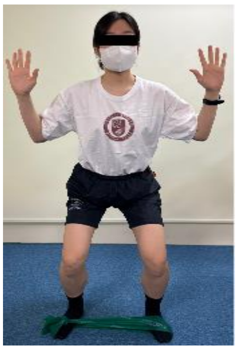

- Sideways walking

- Weeks 1-2: 20 reps, 2 sets, hold for 5 seconds (resistance: green)
- Weeks 3-4: 12 reps, 3 sets, hold for 10 seconds (resistance: green)
- Weeks 5-6: Progression in Theraband resistance (resistance: blue)

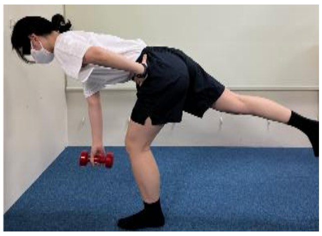

- Single leg Romanian deadlift

- Weeks 1-2: 12 reps, 3 sets
- Weeks 3-4: 12 reps, 3 sets
- Weeks 5-6: additional resistance band applied to the weeks 3-4 (resistance: green)

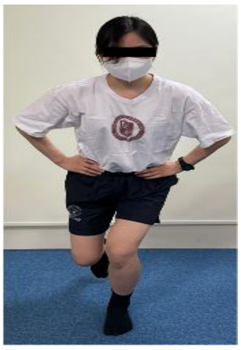

- Single leg standing balance

- Weeks 1-2: 20seconds, 3 sets, with eye opened
- Weeks 3-4: 30seconds, 3 sets, with eye opened
- Weeks 5-6: additional resistance band applied to the weeks 3-4 (resistance: green)
